# First Description of Macrolide-Resistant* Mycoplasma pneumoniae* in Adults with Community-Acquired Pneumonia in Italy

**DOI:** 10.1155/2019/7168949

**Published:** 2019-03-17

**Authors:** Daniela Loconsole, Anna Lisa De Robertis, Rosanna Mallamaci, Anna Sallustio, Anna Morea, Rosa Prato, Michele Quarto, Domenico Martinelli, Maria Chironna

**Affiliations:** ^1^Department of Biomedical Sciences and Human Oncology-Hygiene Section, University of Bari, Piazza G. Cesare 11, 70124 Bari, Italy; ^2^Department of Biosciences, Biotechnology and Biopharmaceutics, University of Bari, Via Orabona 4, 70124 Bari, Italy; ^3^Department of Medical and Surgical Sciences, University of Foggia, Viale Pinto 1, 71122 Foggia, Italy

## Abstract

**Background:**

* Mycoplasma pneumoniae* is a common cause of community-acquired pneumonia (CAP). This cross-sectional study aimed to determine the prevalence of macrolide-resistant* M. pneumoniae* strains in a convenience series of 234 adult hospitalised and nonhospitalised subjects with a diagnosis of CAP in January 2013 to April 2015 in South Italy.

**Methods:**

Respiratory samples were subjected to real-time PCR. In* M. pneumoniae*-positive samples, domain V of 23S rRNA was sequenced to detect resistance-conferring point mutations. P1 major adhesion protein typing and multiple loci variable-number tandem repeat analysis (MLVA) were also performed.

**Results:**

Of the 234 samples, 15 (6.4%) were positive for* M. pneumoniae*. Three of these had a macrolide-resistant genotype: two and one had A2063G and A2064G mutations, respectively. Fourteen of the 15 strains were subtyped: half had subtype 1 and half had subtype 2. Eight strains underwent MLVA profiling: one each had the J, A, and Z type. The remainder was unclassifiable.

**Conclusions:**

This novel discovery of macrolide-resistant* M. pneumoniae* strains in adults with CAP in Italy suggests that there may be increasing circulation of these strains in the population. To facilitate rapid optimization of the antibiotic strategy in Italy, macrolide resistance should be monitored by a surveillance system that is based on molecular methods.

## 1. Introduction

*Mycoplasma pneumoniae* is a leading cause of upper and lower respiratory tract infections in all age groups. It is also a frequent cause of community-acquired pneumonia (CAP).* M. pneumoniae* infections occur endemically and epidemically worldwide. However, the majority of* M. pneumoniae* infections are self-limited, especially in adults, and can be treated with antibiotic therapy without pathogen-specific diagnostic testing [1].

In Europe,* M. pneumoniae* may be responsible for approximately 11% of CAP cases [2] and, in Italy, up to 17% of the hospitalised adult cases of CAP [3]. Macrolides are the first choice of antibiotics for the treatment of CAP caused by* M. pneumoniae* in both adults and children [4]. However, the inappropriate or overuse of such drugs has led to the emergence of macrolide-resistant* M. pneumoniae* strains in several countries [4]. In Italy, macrolide-resistant* M. pneumoniae* strains started emerging in children in 2010 [5]. Moreover, a recent study reported the case of a family in Italy in which a clonal macrolide-resistant* M. pneumoniae* strain was transmitted from the index pediatric case to adult family members [6]. However, the prevalence of macrolide-resistant* M. pneumoniae *strains in adult Italians with CAP has not been reported.

This cross-sectional study examined the prevalence of macrolide-resistant* M. pneumoniae* strains in* M. pneumoniae*-positive specimens from hospitalised and nonhospitalised adults with pneumonia in South Italy (Puglia) in January 2013 to April 2015. Multiple-locus variable-number tandem repeat analysis (MLVA) and P1 adhesion protein typing were also performed.

## 2. Materials and Methods

### 2.1. Study Design, Setting, and Participants

Between January 2013 and April 2015, ten general practitioners and pneumologists working in 12 Divisions of Pulmonary and Respiratory Disease across the Puglia region obtained clinical samples from a convenience series of hospitalised and nonhospitalised Puglia-resident adults (≥18 years) who were diagnosed with CAP. In particular, patients were diagnosed with CAP in presence of at least two clinical criteria among new cough or sputum production, fever >38.0°C or hypothermia <36.1°C, chest pain, dyspnoea, tachypnoea, new altered mental status, abnormal lung examination, respiratory failure, leucocytosis (white cell count >10 × 109/L or >15% bands) or leucopenia (white cell count <4.5 × 109/L), C reactive protein value >3 times the upper limit of normal, and hypoxaemia with a partial oxygen pressure <60mm Hg while the patient was breathing room air and in presence of evidence on chest radiography consistent with pneumonia [7]. The general practitioners and pneumologists were from all six provinces in the Puglia region.

### 2.2. Samples Collection

Nasopharyngeal swabs and/or sputum and/or bronchoalveolar lavage were obtained from all subjects diagnosed with CAP. All clinical samples were collected within 24 h of the clinical onset of symptoms.

### 2.3. DNA Extraction and Polymerase Chain Reaction Amplification

All the collected samples were subjected to real-time PCR analysis for the molecular detection of* M. pneumoniae* and other common agents of CAP, namely,* Legionella* spp.,* Chlamydia pneumoniae*,* Streptococcus pneumoniae*,* Haemophilus influenzae, Moraxella catarrhalis*, and* Staphylococcus aureus*. When multiple biological samples were available from the same patient, only the sample that was positive for the detected pathogens was used for analysis. For the real-time PCR, total nucleic acid was extracted from each respiratory specimen by using the MagnaPure LC automated extraction system (Roche Diagnostics, Milan, Italy). A commercial real-time PCR kit (FTD Bacterial pneumoniae CAP, Arrow Diagnostics, Genoa, Italy) was then used.

### 2.4. Identification of Macrolide Resistance Genotype and Subtyping

*M. pneumoniae*-positive samples were tested for the presence of macrolide resistance by PCR and direct amplicon sequencing in the* M. pneumoniae* 23S rRNA gene to detect single nucleotide polymorphisms that are known to associate with resistance to macrolides. The details have been described elsewhere [5]. The detected* M. pneumoniae* strains were also subtyped molecularly on the basis of their point mutations within the gene encoding the P1 major adhesion protein [8]. Strains were classified as type 1 or type2.

### 2.5. Multiple-Locus Variable Number Tandem Repeat Analysis (MLVA)

MLVA was performed on nucleic acid extracts from clinical specimens as described previously [9]. Interpretation of profiles and MLVA type assignment were achieved according to guidelines reported recently [10]. All tests were performed at the Laboratory of Molecular Epidemiology of the Hygiene Unit of Azienda Ospedaliero-Universitaria Policlinico, Bari, Italy.

### 2.6. Ethical Approval

All procedures performed in the study were in accordance with the ethical standards of the institutional and national research committee and with the 1964 Helsinki declaration and its later amendments or comparable ethical standards. Ethical approval was obtained from the Institutional Review Board at the Apulian Regional Observatory for Epidemiology. Informed written consent was obtained from all individuals who provided the specimens.

## 3. Results

In total, 234 hospitalised and nonhospitalised adult patients with CAP who resided in Puglia during the study period provided specimens. Of these 234 specimens, 15 [6.4%, 95% confidence interval (CI): 3.9–10.3] were positive for* M. pneumoniae* on real-time PCR.* S. pneumoniae* was detected in 43% of the patients,* Haemophilus influenzae* in 17%,* Chlamydia pneumoniae* in 1%,* Legionella pneumophila* in 1%, and* Moraxella catarrhalis* in 5%. The 15 patients ranged between 18 and 87 years of age and a third were hospitalised. The patients with* M. pneumoniae* infection did not show coinfections with other pathogens.

Of the 15* M. pneumoniae*-positive strains, three were macrolide-resistant. Two were from male patients who were 32 and 37 years of age. They had the same 23S rRNA mutation, namely, A2063G. One patient was hospitalised. A link between these two patients was not detected. The third case was a 31-year-old man who was not hospitalised and who had the A2064G mutation (Table 1). Whether these patients had received macrolide treatment before sampling was not known.

P1 typing was performed on 14 of the specimens to differentiate between the strains on the basis of the genetic sequence that encodes the P1 adhesion molecule. One sample was not available for P1 typing. Seven strains were type 1 strains while the remaining seven strains were type 2 strains.

Eight* M. pneumoniae* strains were characterized by MLVA. The remaining 7 strains were not characterized due to insufficiency of clinical samples. Multiple MLVA types were detected. In three cases (patients Nos. 4, 7, and 10 of Table 1), the MLVA types were J, A, and Z: all three types have been identified previously. In the other five cases, the strains were untypeable when using the scheme proposed by Degrangè* et al*. [9].

## 4. Discussion

The increasing resistance of* M. pneumoniae* strains to macrolides has become a worrisome health problem in the last few decades.

In the present study, the overall prevalence of* M. pneumoniae* in adult Italian patients with CAP was 6.4%. Macrolide-resistant strains were found in the 1.3% of subjects.

In Italy, macrolide-resistant* M. pneumoniae* was first reported in pediatric patients in an outbreak of* M. pneumoniae* in 2010 [5] and only sporadically in Italian adults [6].

At present, there is relatively little information in the literature on macrolide-resistant* M. pneumoniae *in adults in Europe. Macrolide-resistant* M. pneumoniae* at very low level was detected in a study conducted on a population of wide age range (15–65-year-old patients) in England and Wales [11].

Recently, a German study showed that, in 2011–2012, the 3.1% of adults with CAP had macrolide-resistant* M. pneumoniae* strains [12]. Our novel findings thus suggest that there is a spread in adults, although at low level, of macrolide resistance of* M. pneumoniae* also in Italy at present.

In our study, the most frequent mutation in domain V of the 23S rRNA gene was the A2063G mutation: two of the three patients with macrolide-resistant* M. pneumoniae* had this mutation. The remaining patient bore the A20164G mutation. All of these mutations are known to confer strong resistance to macrolides [13]. This presence of macrolide resistance may be the consequence of the frequent use of macrolides in Italy, especially for CAP in home-treated patients; this may have placed selective pressure on* M. pneumoniae* strains [14, 15]. We also observed that the* M. pneumoniae* strains we detected were equally likely to be P1 gene type 1 and type 2. By contrast, in the outbreak of* M. pneumoniae* in 2010 among Italian children, the P1 subtype predominated [5]. This disparity may reflect the fact that the latter cases were detected in an epidemic (where one subtype may be more likely to predominate), whereas our cases were not epidemiologically linked. No correlation emerged between macrolide resistance and other molecular characteristics of MP strains (P types and MLVA profiles).

MLVA typing of our* M. pneumoniae* strains revealed the J, A, and Z type [9] in one patient each. The remaining five strains that were tested were not classifiable. This indicates the heterogeneity of the MLVA profiles of the circulating strains. As is known, locus MPN1 is localized in the variable hsdS gene that changes constantly under the environmental pressure and variations in this locus do not necessarily represent emerging of a new the strain [15]. If the MPN1 allele profile is excluded from the classification, as was proposed by some authors [15], the most frequent profile in our samples was 4/5/7/2, which was detected in three cases. This MLVA profile frequency is in accordance with the frequency of MLVA profiles reported by other studies on adults with CAP due to* M. pneumoniae *[15, 16]. Nevertheless, we believe that the MPN1 allele sequence helps to assess the relationships between* M. pneumoniae* strains and we still recommend the method of Degrangè et al. [9]. On the other hand, a more discriminative scheme is needed for epidemiological purposes [17]. Whole-genome sequencing of* M. pneumoniae* could help in the future to improve such analyses.

European guidelines indicate that macrolides should be the first choice treatment of* M. pneumoniae* infections in both children and adults [18]. However, if the fever persists 48–72 h after starting this first-line drug, it is recommended to change treatment to second-line antibiotics [19]. It is important to preserve the efficacy of macrolides for* M. pneumoniae* infections and other antibiotics while we wait for the development of new antibiotic agents that have an acceptable safety profile in all age groups. The best way to do this is to take into account the evidence that shows that macrolide-resistant* M. pneumoniae* are circulating and to advance antibiotic stewardship initiatives. These initiatives are particularly important in countries such as Italy, which is one of the European countries that have serious problems with antibiotic resistance [20].

The study has some limitations. First, the number of CAP cases positive for* M. pneumoniae* was relatively low; this may be due to the fact that samples were collected during an interepidemic phase. Further studies should be undertaken during the epidemic phase to estimate the actual prevalence of macrolide-resistant* M. pneumoniae* strains. Second, the results of our study should be interpreted with caution due to possible selection bias caused by the sampling method.

Finally, our study shows that strict surveillance and monitoring are needed to manage the emerging resistance to first-line therapy for* M. pneumoniae* in adults in Italy.

## 5. Conclusions

We found macrolide-resistant* M. pneumoniae* strains in hospitalised and nonhospitalised adult patients with CAP. In all cases, the mutations identified in domain V of the 23S rRNA gene conferred strong resistance to macrolides. Characterization of* M. pneumoniae* strains by P1 major adhesion protein gene typing and MLVA revealed the circulation of very heterogeneous strains. To facilitate rapid optimization of the antibiotic strategy in Italy, macrolide resistance should be monitored by a surveillance system that is based on molecular methods.

## Figures and Tables

**Table 1:** Findings of the 15 patients living in the Puglia region of Italy whose swab, sputum, or BAL samples were positive for *M. pneumoniae* in January 2013 to April 2015.

Patient no.	Age, years (sex)	Sample type	Hospitalized	Susceptibility to macrolides (SNP)	MLVA type	MLVA profile	P1 gene type
1	65 (F)	BAL^a^	yes	wt	-	-	1
2	80 (M)	sputum	no	wt	-	-	2
3	87 (F)	sputum	no	wt	-	-	-
4	44 (M)	BAL^a^	yes	wt	J	(3/4/5/7/2)	1
5	32 (M)	NPS^b^	no	R (A2063G)	NC^c^	(3/0/6/6/2)	2
6	62 (M)	NPS	no	wt	NC^c^	(0/0/5/6/2)	2
7	61 (F)	NPS	no	wt	A	(1/4/5/7/2)	1
8	20 (M)	sputum	yes	wt	NC^c^	(5/0/5/6/2)	2
9	49 (M)	NPS	no	wt	NC^c^	(7/0/6/5/2)	2
10	37 (F)	BAL^c^	yes	R (A2063G)	Z	(7/4/5/7/2)	1
11	37 (F)	NPS	no	wt	-	-	1
12	18 (M)	NPS	yes	wt	-	-	1
13	52 (M)	NPS	no	wt	-	-	2
14	23 (F)	sputum	no	wt	NC^c^	(3/0/6/7/2)	1
15	31 (F)	NPS	no	R (A2064G)	-	-	2

^a^ Bronchoalveolar Lavage.

^b^ Nasopharyngeal swab.

^c^ Not classifiable using the scheme proposed by Degrangè et al. (2009).

MLVA: multiple-locus variable-number tandem repeat analysis; R: resistant; SNP: single nucleotide polymorphism; wt: wild type.

## Data Availability

The data used to support the findings of this study are available from the corresponding author upon request.

## Abbreviations

*M. pneumoniae*: * Mycoplasma pneumoniae*
CAP: Community-acquired pneumonia
MLVA: Multiple-locus variable-number tandem repeat analysis.
